# Adeno-associated virus delivery of anti-alpha toxin monoclonal antibodies confers protection against *Staphylococcus aureus* infections

**DOI:** 10.1371/journal.ppat.1014090

**Published:** 2026-04-06

**Authors:** Josefien W. Hommes, Madison E. Hughes, Derek Cheung, Björn Petri, Lindsey M. Orthner, Tristan J. van der Linden, Bart W. Bardoel, Simran K. Deo, Deepak T. Patel, Ronald S. Flannagan, David E. Heinrichs, Sarah K. Wootton, Bas G. J. Surewaard

**Affiliations:** 1 Department of Microbiology, Immunology, and Infectious Disease, Snyder Institute for Chronic Diseases, University of Calgary, Calgary, Canada; 2 Department of Pathobiology, University of Guelph, Guelph, Canada; 3 Department of Medical Microbiology, University Medical Center Utrecht, Utrecht University, Utrecht, Netherlands; 4 Department of Microbiology and Immunology, The University of Western Ontario, London, Canada; University of Illinois at Chicago College of Medicine, UNITED STATES OF AMERICA

## Abstract

*Staphylococcus aureus* is a major cause of infections, ranging from mild skin conditions to severe life-threatening systemic diseases. Despite decades of effort, vaccine development has been unsuccessful, highlighting the need for alternative approaches. One promising candidate is MEDI4893 (Suvratoxumab), a monoclonal antibody that neutralizes α-toxin (AT), a key virulence factor with diverse functions including cellular lysis and induction of platelet aggregation during sepsis. MEDI4893 has demonstrated efficacy in various preclinical models of infection and showed encouraging results in a phase IIb clinical trial for ventilator-associated pneumonia. However, as an exogenous antibody, MEDI4893 is limited by its short half-life, high production costs, and restricted availability. To overcome these challenges, we investigated vectored immunoprophylaxis as a novel strategy for long-term protection. This approach uses an adeno-associated viral vector (AAV) to deliver the MEDI4893 gene, enabling sustained *in vivo* antibody expression. Following AAV-MEDI4893 administration, mice developed high and stable antibody levels in plasma and detectable titers at mucosal surfaces. Using intravital microscopy, we observed that vector-mediated MEDI4893 effectively prevented AT-induced platelet aggregation and microvascular thrombosis, thereby protecting animals from the lethal effects of intravenous toxin challenge. Infection studies confirmed that this protective effect extended to clinically relevant models. In murine pneumonia models, treated mice showed improved survival and reduced sickness behavior despite similar bacterial burdens, while in skin infection models, they were protected from dermal necrosis and exhibited lower bacterial loads. These findings highlight that AT is a major driver of pathology across multiple tissues and that its neutralization can mitigate disease severity. Together, our results demonstrate that AAV-mediated delivery of MEDI4893 provides durable, protective antibody levels and effectively neutralizes AT *in vivo*. This strategy represents a cost-effective, long-lasting alternative to traditional monoclonal antibody therapy and offers a promising prophylactic approach to mitigate *S. aureus* infections in the absence of a vaccine.

## Author summary

*Staphylococcus aureus* is a major cause of infections worldwide, ranging from common skin infections to life-threatening pneumonia and sepsis. With no effective vaccine and increasingly common antibiotic resistance, these infections are notably difficult to prevent and treat. One of the most harmful weapons used by *S. aureus* is α-toxin (AT), which damages cells, weakens protective barriers, and causes dangerous blood clotting that can lead to organ failure. MEDI4893 (Suvratoxumab), an antibody that blocks AT, has shown protective effects in both animal studies and early human trials. However, traditional antibody treatments are expensive, short-lived, and require repeated administration. In this study, we tested a new approach called vectored immunoprophylaxis (VIP). We used a harmless virus (AAV) to deliver the MEDI4893 gene, allowing the body to make the antibody itself for long-term protection. Treated mice maintained high antibody levels, were protected from lethal toxin exposure, and lung and skin infections were mitigated. These results suggest that VIP could provide lasting protection for patients most at risk, including those with weakened immune systems, offering a promising alternative where vaccines and antibiotics fall short.

## Introduction

*Staphylococcus aureus* (*S. aureus*) is a Gram-positive opportunistic pathogen that colonizes the human skin and nasal cavity. While often harmless as a commensal, it can cause severe invasive infections of the skin, soft tissues, and the lung, as well as bloodstream infections, vegetations on heart valves, endocarditis, and osteomyelitis [1]. Its ability to adapt and thrive in diverse environments, combined with a repertoire of virulence factors, makes it a leading cause of infection-related morbidity and mortality globally [2,3]. The worldwide emergence of methicillin-resistant *S. aureus* (MRSA) strains resistant to first-line antibiotics has substantially compromised the effectiveness of therapeutic interventions. Strikingly, high rates of treatment failure persist even when appropriate antibiotic regimens are administered at concentrations well above established clinical susceptibility breakpoints [4]. These limitations have led to the hypothesis that anti-virulence strategies may represent an attractive therapeutic alternative, as they aim to mitigate host pathology while preserving the beneficial commensal microbiota [5,6]. One attractive anti-virulence drug target is α-toxin (AT or α-hemolysin), a 33 kDa exotoxin secreted by *S. aureus* that plays a central role in the pathogenesis of infection [7,8]. AT is highly conserved among clinical *S. aureus* isolates, and its expression correlates with disease severity [9]. Additionally, hospital isolates of *S. aureus* that harbor defective AT showed reduced virulence [10]. AT binds to host cells via the surface protein receptor A Disintegrin and Metalloproteinase 10 (ADAM10), where it oligomerizes into transmembrane pores that cause cell lysis of many cell types, including endothelial cells and leukocytes, leading to tissue destruction [11,12]. Beyond direct toxicity, AT has been shown to promote immune dysregulation and increase vascular permeability [13–15]. At sub-lytic concentrations, AT further activates ADAM10, triggering cleavage of junctional and adhesion molecules and initiating proinflammatory signaling cascades that exacerbate tissue injury [16]. Previously, our lab has shown that AT binding to the ADAM10 receptor mediates severe dysfunction of platelets [17]. AT release induces platelet aggregation, producing microthrombi that occlude small blood vessels throughout the body, impairing the function of many organs.

Given the central role of AT in tissue pathology during *S. aureus* infection, neutralizing this toxin using monoclonal antibody (mAb) therapy or small molecules could be a promising alternative to traditional antibiotics [18]. This approach is further supported by observations that naturally acquired anti-AT neutralizing antibody levels in human populations are variable and often relatively low, typically in the range of approximately 1–5 µg/mL, with substantial inter-individual variability. Notably, lower levels have been reported in individuals at higher risk of severe *S. aureus* infection, underscoring the potential need for therapeutic augmentation [19,20]. The mAb MEDI4893/AR-320 (Suvratoxumab) is a human IgG that targets a highly conserved epitope of AT, present in more than 97% of clinically isolated *S. aureus* strains [21]. A phase IIb clinical trial of intravenous MEDI4893 infusion was recently completed, revealing promising efficacy against ventilator-associated pneumonia in a particular subgroup of patients [8]. Another anti-AT antibody, AR-301, is also currently advancing into a phase III clinical trial for the treatment of staphylococcal pneumonia (NCT03816956).

MAb therapy offers a promising strategy to circumvent antibiotic resistance; however, there remain several disadvantages: the half-life of exogenous mAbs is short, high dosages are required, and production is costly. Vectored immunoprophylaxis (VIP) addresses these limitations by employing adeno-associated virus (AAV) vectors to deliver genes encoding mAbs [22]. AAV is a non-enveloped, single-stranded DNA virus that is apathogenic and relies on co-infection with a helper virus, such as adenovirus, for replication and production of progeny virions. Wildtype AAV contains a Rep and Cap gene surrounded by inverted terminal repeat (ITR) sequences, while recombinant AAV (rAAV) is a modified form of AAV where the Rep and Cap genes are replaced by a transgene of interest. The use of these vectors to deliver transgenes encoding mAbs has emerged as an effective technique for providing sustained *in vivo* mAb expression. VIP has already shown robust protective efficacy against a wide range of infectious diseases including human immunodeficiency virus (HIV), influenza virus, Ebola virus, Marburg virus and *Clostridium difficile* across several different animal models [22–25] and has been investigated in two human clinical trials against HIV (NCT01937455, NCT03374202) [26,27].

In this study, we use VIP technology to deliver the humanized MEDI4893 gene into mice and investigate how this approach mediates protection against *S. aureus* infection. Using spinning-disk intravital microscopy (SD-IVM), we demonstrate that VIP completely prevents platelet aggregation following AT challenge by preventing the interaction between AT and platelets. Collectively, our results show promise for AAV-mediated MEDI4893 delivery as an alternative approach to *S. aureus* infection treatment.

## Results

### Analysis of AAV6.2FF-mediated expression of MEDI4893 in mice

**Fig 1:**
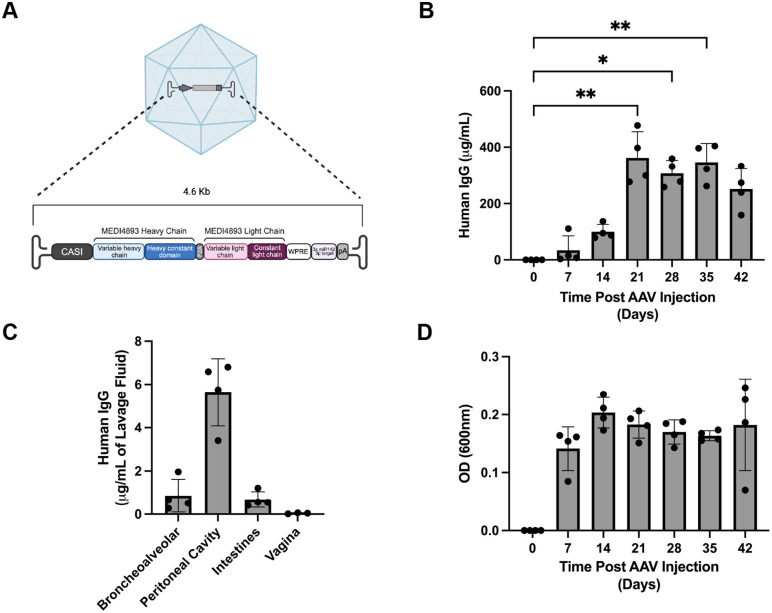
AAV-MEDI4893 expression kinetics in blood and mucosal tissues of transduced mice and conformation of binding to α-toxin. **(A)** Schematic diagram of AAV-MEDI4893 genome. Transgene expression is driven by the ubiquitous CASI promotor, followed by the human MEDI4893 variable heavy chain domain and heavy chain constant domain, a furin F2A self-cleaving peptide, the variable light chain domain and light chain constant domain of MEDI4893. This was followed by a Woodchuck Hepatitis Virus Posttranscriptional Regulatory Element (WPRE) and 3x miR142 3p target and a simian virus 40 polyadenylation signal (SV40 polyA), flanked by two AAV2 inverted terminal repeat (ITR) sequences. Genome length from ITR to ITR is 4587 base pairs. Schematic created in BioRender. Hommes, J. (2026) https://BioRender.com/ibh05k0. **(B)** Plasma expression kinetics of human IgG following AAV-MEDI4893 administration in 6-week-old Balb/c mice (*n* = 4/group). Blood was collected up to 42 days post-injection, and mAb concentrations were determined by human IgG ELISA. Kruskal-Wallis test; * p < 0.05, ** p < 0.01; mean + SD. **(C)** Human IgG concentrations measured in bronchoalveolar lavage, peritoneal lavage, intestinal lavage, and vaginal lavage fluids at day 42 post-AAV administration (n = 4 for all groups except n = 3 for vaginal lavage; mean +/- SD). Antibody levels quantified by human IgG ELISA. **(D)** Functional binding of AAV-expressed MEDI4893 human IgG in mouse plasma to recombinant AT, measured by indirect ELISA at indicated timepoints post-AAV-MEDI4893 administration (*n* = 4 per group; mean +/- SD).

MEDI4893 was originally derived from VelocImmune humanized mice and has been extensively validated for its ability to neutralize AT toxicity in diverse experimental models [28–30]. The MEDI4893 genes were human-codon-optimized and synthesized with human IgG1 heavy chain and human kappa light chain constant domains separated by an F2A self-cleaving peptide see (**Fig 1A**) for schematic. For *in vivo* delivery, AAV-MEDI4893 vectors were generated and quantified using real-time PCR. Balb/c mice were administered the AAV-MEDI4893 vector intramuscularly (IM) at a dose of 1x10^11^ vector genomes, as this approach is well established to achieve high and sustained systemic antibody titers following a single administration [22,31]. Murine plasma concentrations of MEDI4893 were quantified over 42 days using a human IgG sandwich ELISA (**Fig 1B**). Peak expression (362 µg/mL ± 92) was observed 21 days after AAV administration, after which MEDI4893 levels remained persistently high (300–400 µg/mL) throughout the study period. The plasma levels of MEDI4893 were in line with what was previously observed for other AAV-mAbs [22,31]. In addition to plasma concentrations, levels of MEDI4893 at mucosal surfaces of the lungs, peritoneum, intestines, and vagina were determined at 42 days post-AAV-MEDI4893 administration (**Fig 1C**). Antibody concentrations were highest in the peritoneal cavity and were also detectable at other mucosal surfaces, although at substantially lower levels than in the blood. To confirm functional binding of AAV-expressed MEDI4893 to AT, ELISA plates were coated with recombinant AT and incubated with plasma collected from mice at various time points post-AAV-MEDI4893 treatment (**Fig 1D**). High levels of AT-binding MEDI4893 were detectable as early as 7 days after AAV-MEDI4893 and remained high over the duration of the experiment. Mouse serum from AAV-MEDI4893-treated animals was able to rescue AT-induced cell death of type 2 alveolar-like epithelial cells (A594 cell-line) compared to control serum (**S1 Fig**). To control for potential off-target effects of the AAV vector, we included an AAV expressing a non- *S. aureus* specific isotype control antibody. Specifically, we used AAV-CA45 [32], which encodes a human IgG1 directed against Ebola virus and therefore lacks specificity for *S. aureus*. Together, these results show that IM administration of AAV-MEDI4893 mediates robust and sustained expression of functional human anti-AT mAbs that can be detected in plasma and mucosal surfaces.

### AAV-MEDI4893 delivery confers protection against AT challenge in vivo

**Fig 2:**
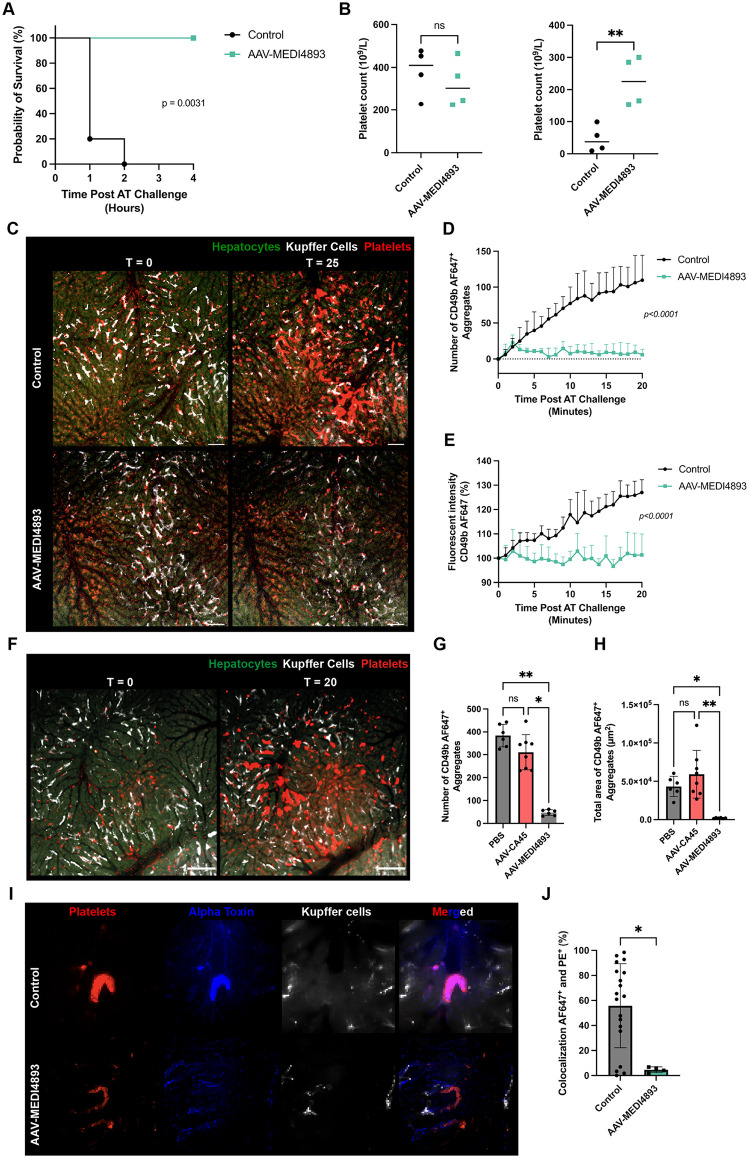
AAV-MEDI4893 administration prevents platelet aggregation and mortality following α-toxin infusion. **(A)** Survival of untreated control and AAV-MEDI4893-treated mice following AT intoxication. (*n* = 4 per group); Kaplan-Meier survival analysis with Mantel-Cox test. **(B)** Quantification of blood platelet levels at baseline (left) and 10 min post-AT infusion (right). (*n* = 4 per group); unpaired t test, ** p < 0.01; median. **(C)** Spinning-disk intravital microscopy (SD-IVM images of the liver before and 20 min after AT infusion in control or AAV-MEDI4893 treated C57Bl/6 mice. Images correspond to S1-S2 Videos. Platelets (CD49b; red). Liver resident Kupffer cells (TIM-4; white), and hepatocytes (auto-fluorescent, dull green). **(D)** Quantification of platelet aggregates (area> than 5-50 µm2) and **(E)** platelet fluorescent intensity (CD49b) from SD-IVM videos shown in **(C)**. (*n* = 3 per group); unpaired t-tests; mean + SE. Scale bar 50 µm, **(F)** Spinning-disk intravital microscopy (SD-IVM images of the liver before and 20 min after AT infusion in AAV-CA45 treated C57Bl/6 mice. Platelets (CD49b; red). Liver resident Kupffer cells (TIM-4; white), and hepatocytes (auto-fluorescent, dull green); Scale bar 100 µm. **(G)** Quantification of platelet aggregates (area> than 5 µm2) and **(H)** total area covered by platelets after 20 minutes of AT injection in PBS control mice, AAV-CA45-, or AAV-MEDI4893-treated mice. (*n* = 5 for PBS, *n* = 8 for AAV-CA45, and *n* = 6 for AAV-MEDI4893). Kruskal-Wallis test, * p < 0.05; ** p < 0.01; mean +/- SD. **(I)** High-magnification SD-IVM images showing colocalization of N-terminally labeled AT (AT-AF647; blue) with platelet aggregates (CD49b; red) 15 minutes post-infusion in control (top) or AAV-MEDI4893-treated mice (bottom). Platelet aggregates (CD49b; red), Kupffer cells (F4/80; white) **(J)** Quantification of colocalization of platelet aggregates with AT from images in (I) (*n* = 19 aggregates for control and *n* = 4 aggregates for AAV-MEDI4893 treated animals). Experiment was repeated twice. Mann-Whitney test, * p < 0.05; mean +/- SD.

We previously reported that prophylactic administration of purified MEDI4893 protects against the detrimental effects of AT on platelets, prevents organ damage, and reduces AT-induced mortality [17]. In those experiments, a dose of 30 mg/kg was used, corresponding to approximately 400 µg/mL of antibody in mouse blood, a concentration consistent with the expression levels achievable using vectorized MEDI4893 (**Fig 1B**). We next sought to determine whether AAV-mediated delivery of MEDI4893 could confer resistance to AT in mice. Given the profound detrimental effects of AT on mouse physiology, infusion of 1 µg AT into the bloodstream of anesthetized control mice resulted in rapid mortality within one hour. In contrast, AAV-MEDI4893–treated mice were completely protected and survived for the full 4-hour duration of the experiment (Fig 2A). Moreover, to assess whether AAV-MEDI4893 prevented AT-induced platelet aggregation, platelet counts were quantified before AT infusion and 10 minutes after AT intoxication. Control animals exhibited a marked reduction in circulating platelets, whereas AAV-MEDI4893–treated mice maintained significantly higher platelet counts (Fig 2B). Although a modest decline in platelet numbers was observed in AAV-MEDI4893-treated animals, the decrease was substantially less pronounced than in untreated controls (**S2 Fig**). Together, these results demonstrate that AAV-mediated delivery of MEDI4893 provides robust protection against the lethal effects of systemic AT intoxication and significantly preserves circulating platelet counts.

Next, we employed intravital microscopy to investigate the intravascular and cellular events triggered by AT intoxication in the bloodstream. Using this approach, we previously demonstrated that AT infusion induces widespread, irreversible platelet aggregation throughout the body in multiple vascular beds, with the majority of aggregates becoming sequestered in the liver [17]. To assess whether AAV-MEDI4893 prevents this process, we performed real-time SD-IVM. Untreated control or AAV-MEDI4893-treated mice were imaged, and AT was administered one minute after video acquisition started. In control mice, extensive platelet aggregation was observed starting at 7–9 min after AT infusion (**S1 Video**). Conversely, no platelet aggregation was detected in AAV-MEDI4893-treated animals during the same timeframe (**S2 Video** and **Fig 2C**). Consistent with this, treated mice exhibited markedly fewer aggregates (**Fig 2D**) and decreased fluorescent platelet signal intensity (**Fig 2E**). As shown in Fig 2F**-**2H, AAV-CA45-treated mice exhibited platelet aggregation comparable to PBS controls following AT administration, indicating that the vector itself does not confer protection. These findings confirm that the inhibition of AT-induced platelet aggregation is specifically mediated by MEDI4893 expression rather than by nonspecific effects of AAV delivery. Platelets express the AT receptor ADAM10 and can therefore be directly targeted by AT, which induces platelet aggregation upon binding [16,17]. To assess whether vectorized MEDI4893 disrupts the AT-platelet interaction, we infused fluorescently labeled AT (AT-AF647) and monitored its direct binding to platelets *in vivo*. Following infusion of AT-AF647, control animals exhibited an initial burst of fluorescence, followed by rapid platelet aggregation and accumulation of AT within the aggregates (**S3 Video**). High-magnification imaging of control animals revealed strong colocalization of AT with platelet aggregates (Fig 2I-2J). In contrast, no colocalization was observed in AAV-MEDI4893-treated animals. Even in the few aggregates that formed, AT binding was absent, and the platelet clusters were not covered with toxin. Collectively, these findings demonstrate that AAV-MEDI4893 treatment prevents AT from binding to ADAM10 on platelets, thereby protecting mice from the deleterious effects of intravenous AT challenge.

### AAV-MEDI4893 treatment protects against staphylococcal lung- and skin-infection

**Fig 3:**
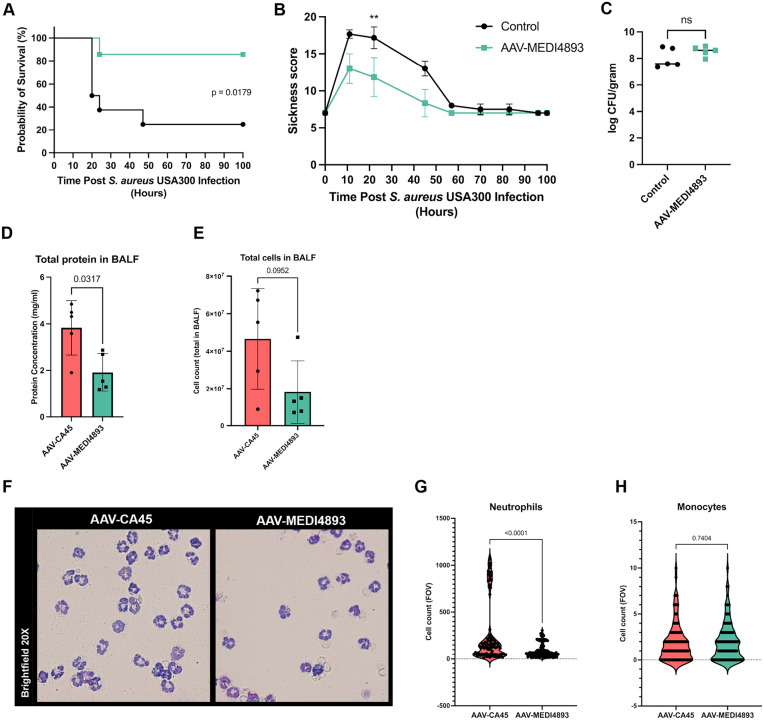
Effect of AAV-MEDI4893 treatment on *S. aureus* lung infection model. **(A)** Survival of mice receiving a lethal dose of *S. aureus* (5x10^8^ CFU USA300 LAC) in Control or AAV-MEDI4893-treated groups over a period of 4 days post-infection (*n* = 8 for PBS control mice and *n* = 7 for AAV-MEDI4893 mice). Survival was tested with Kaplan-Meier with Mantel-Cox test. **(B)** Sickness score of the same cohorts was assessed over 4 days post-infection using the standardized sepsis scoring system (see S1 Table), Mann-Whitney tests, ** p < 0.01; mean + /- SD. **(C)** Bacterial burden in lung tissue at 24 hours post intratracheal infection with *S. aureus* (5x10^8^ CFU USA300 LAC), (*n* = 5 per group), Mann-Whitney test; median. **(D)** Total BAL fluid protein concentrations from mice treated with AAV-CA45 or AAV-MEDI4893 (*n* = 5) Mann-Whitney test, mean + /- SD. **(E)** Number of total cells collected from BALF of mice treated with AAV-CA45 and AAV-MEDI4893 (*n* = 5). Mann-Whitney test, mean + /- SD. **(F)** Representative picture of hemacolor stained cytospins of BALF of mice treated with AAV-CA45 or AAV-MEDI4893. Quantification of **(G)** neutrophils and **(H)** monocytes in the bronchoalveolar lavage fluid (BALF) of mice treated with AAV-CA45 and AAV-MEDI4893 after 24 hours of intratracheal infection with a lethal dose of *S. aureus* (*n* = 5). Mann-Whitney tests, mean + /- SD.

Although AT is a major virulence factor, it represents only one of many toxins and virulence factors produced by *S. aureus* and is therefore not solely responsible for infection-associated pathology. Having established that AAV-MEDI4893 confers protection against a lethal dose of AT intoxication, we next investigated whether it could also provide protection in more clinically relevant models of infection, including skin and lung disease. To this end, we employed two different *S. aureus* infection models to further evaluate the efficacy of AAV-MEDI4893 treatment. AT has previously been shown to play a critical role in *S. aureus* pneumonia, which is also the primary indication currently being investigated in the ongoing phase III clinical trial of anti-AT antibody therapy [33,34]. Therefore, we exposed mice to intratracheal infection with *S. aureus* USA300 LAC for a period of four days. Body weight, mortality and sickness behavior were monitored, with the latter assessed using the standardized sepsis scoring system (**S1 Table**). Mice treated three weeks prior with AAV-MEDI4893 were significantly more likely to survive this infection compared to healthy controls (**Fig 3A**). While no significant differences in bodyweight were observed between the two groups (**S3 Fig**), sickness scores were significantly lower in treated mice at 24 hours post-infection (**Fig 3B**). At later time points, the difference in sickness behavior diminished due to mortality in the control group, which reduced the number of animals available for scoring. Importantly, the survival benefit was not associated with differences in bacterial burden, as lung colony-forming units (CFUs) were similar after 24 hours (**Fig 3C**). In a separate set of experiments, we performed intratracheal infections in mice treated with either AAV-MEDI4893 or the control AAV-CA45 vector. Analysis of bronchoalveolar lavage fluid (BALF) revealed increased total protein levels in AAV-MEDI4893-treated mice (**Figs 3D and** **S4**), along with a trend towards increased total cell numbers (**Fig 3E**), consistent with altered pulmonary vascular permeability and immune cell recruitment. Despite these changes, bacterial dissemination to the blood, liver, kidney and spleen remained comparable between groups (**S5 Fig**), indicating that AAV-MEDI4893 does not impair bacterial establishment or spread. Importantly, quantitative analysis of BAL cytospins demonstrated a significant reduction in neutrophil infiltration in AAV-MEDI4893-treated mice compared to AAV-CA45 controls, while monocyte numbers were unchanged (Fig 3F–3H). Together, these findings indicate that AAV-MEDI4893 mitigates the inflammatory response during *S. aureus* USA300 LAC pneumonia particularly by limiting neutrophil-driven inflammation without affecting bacterial burden, supporting a model in which toxin neutralization reduces immunopathology rather than bacterial load.

**Fig 4:**
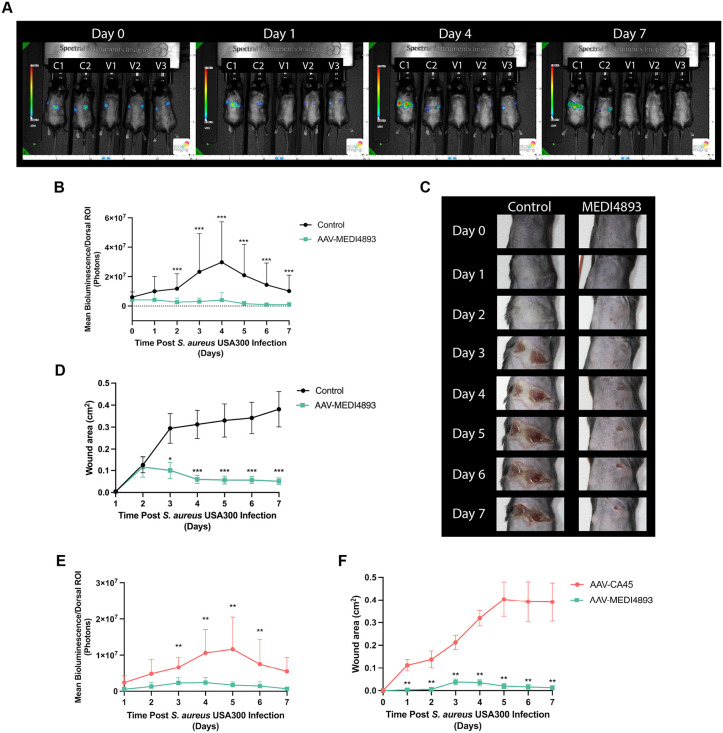
Effect of AAV-MEDI4893 treatment on *S. aureus* skin infection model. **(A)** Whole-body bioluminescence imaging of mice bilaterally infected intradermally with *S. aureus* (2x10^7^ CFU, USA300 LAC-lux per site) at day 0, day 1, day 4, and day 7 post-infection. Control mice (left two) and AAV-MEDI4893-treated mice (right three) were imaged in parallel. **(B)** Quantification of bioluminescence signals from control mice (*n* = 14) and AAV-MEDI4893-treated mice (*n* = 12), Multiple Mann-Whitney tests, *** p < 0.001; mean + SD. **(C)** Daily photographs of one representative untreated control mouse and one AAV-MEDI4893-treated mouse to monitor dermal necrosis at sites of infection. **(D)** Quantification of dermal necrosis from panel (C) for control (*n* = 14 mice) and AAV-MEDI4893-treated mice (*n* = 12), Multiple Mann-Whitney tests, * p < 0.05, *** p < 0.001; mean + /- SEM. **(E)** Quantification of bioluminescence signals from mice treated with AAV-CA45 (*n* = 6) and AAV-MEDI4893 (*n* = 6), Multiple Mann-Whitney tests, ** p < 0.01; mean + SD. **(F)** Quantification of dermal necrosis for mice treated with AAV-CA45 (*n* = 6) and AAV-MEDI4893 (*n* = 6), Multiple Mann-Whitney tests, ** p < 0.01; mean + /- SEM.

In addition to its role in staphylococcal pneumonia, AT is also a critical determinant of disease severity during skin and soft tissue infections (SSTIs) [35]. Both the severity of primary SSTIs as well as recurrence of these infections are related to the expression of AT. Targeting either the toxin or its receptor, ADAM10, has been shown to improve outcomes and decrease recurrence rates [35]. Building on our findings that AAV-MEDI4893 protects against AT-mediated pathology in pneumonia, we next assessed its efficacy in a model of *S. aureus* skin infection. Mice treated with AAV-MEDI4893 or untreated were challenged bilaterally with luminescent *S. aureus* USA300 LAC and monitored over a period of seven days. Noninvasive whole-body bioluminescence imaging revealed a significant decrease in bacterial burden in treated animals compared to controls (Fig 4A**-**4B). Moreover, while control mice developed large areas of dermal necrosis at the site of infection, AAV-MEDI4893-treated animals were protected from tissue destruction (Fig 4C**-**4D). In a separate set of experiments directly comparing AAV-MEDI4893 to the control vector AAV-CA45, similar results were observed. Mice receiving AAV-MEDI4893 showed a significant reduction in bacterial burden, as measured by bioluminescent signal (**Fig 4E**), along with markedly reduced lesion size and dermal necrosis compared to AAV-CA45-treated controls (**Fig 4F**). These findings further confirm that the protective effect is specific to MEDI4893-mediated neutralization of AT. Together with our results, these studies highlight that AT not only drives local tissue damage but also facilitates bacterial persistence and expansion within the skin. Furthermore, they demonstrate that AAV-mediated delivery of MEDI4893 provides durable protection against AT-driven pathology in both pneumonia and skin infection models. By protecting against two major clinical manifestations of *S. aureus* disease, this work underscores the broad potential of AAV-based anti-virulence strategies as a novel therapeutic approach to combat antibiotic-resistant staphylococcal infections.

## Discussion

In this study, we demonstrated that a vector-based genetic delivery of monoclonal human IgG MEDI4893 directed against *S. aureus* AT provides durable protection against both purified toxin challenge and clinically relevant models of infection. Our lab has previously reported that prophylactic administration of purified MEDI4893 protected against AT intoxication and had partial efficacy in a *S. aureus* bloodstream infection model [17]. However, the clinical application of mAb therapy is constrained by its relatively short half-life, limiting its utility as a long-term preventive strategy. AAV-mediated antibody delivery addresses this limitation by enabling sustained *in vivo* expression from a single administration. Consistent with prior reports, including long-term expression exceeding 1071 days in sheep [36] and persistent antibody levels in mice [31,37], we we observed stable circulating MEDI4893 levels for at least 42 days. Importantly. the antibody concentrations achieved in our model (~300–400 µg/mL) are comparable to or exceed those reported in clinical settings. In the phase IIb SAATELLITE trial, a single 5 g dose of suvratoxumab resulted in mean serum levels of ~296 µg/mL at 30 days, with most patients remaining above a ~ 211 µg/mL target [8], although protection was only observed in a subset of patients and antibody levels declined over time. In contrast, AAV-mediated delivery maintained similar or higher concentrations over extended periods. Nevertheless, clinical efficacy may depend on host factors; for example, recent work suggests that reduced expression of the AT receptor ADAM10 in elderly individuals may limit the benefit of anti-AT strategies, consistent with diminished protection in aged versus young mice [38]. In addition, both the duration of expression and the achieved antibody titers were substantially greater than those obtained with mRNA delivery of MEDI4893, which was recently reported to confer only short-term protection [39]. Taken together, these findings strongly suggest that AAV-based delivery can achieve prolonged antibody expression at clinically relevant levels provide lasting protection against toxin-mediated staphylococcal disease.

Vector delivery has proven effective in multiple infectious disease models and is currently under evaluation in two HIV prevention trials (NCT03374202, NCT01937455) underscoring its translational potential. Several features make vectorized prophylaxis particularly attractive: vectors can be engineered to evade pre-existing immunity against wild-type AAV, and host cell tropism can be directed through capsid adjustments [40]. Moreover, by driving direct transgene expression of antibodies, this strategy circumvents the need for an intact adaptive immune response, meaning T and B cells are not necessary for antibody induction. This feature is particularly advantageous for immunocompromised patients, in whom conventional vaccination often fails to elicit protective immunity. Indeed, AAV does not replicate in the absence of helper viruses and has low immunogenicity, which supports its use in vulnerable populations. In immunocompromised individuals, however, there are theoretical considerations, including altered vector clearance, prolonged transgene expression, or rare risks of vector-related toxicity [41]. However, to date, clinical data have not identified significant safety concerns specifically associated with AAV administration in these populations, including with comorbid conditions such as HIV infection [42]. The need is especially acute in the context of *S. aureus* infections, where no licensed vaccine exists and antibiotic resistance increasingly limits treatment options. Patients with inborn errors of immunity, such as chronic granulomatous disease (CGD) or OTULIN haploinsufficiency [43,44], are at high risk of recurrent and severe staphylococcal disease and are particularly prone to damage by AT. Consistent with this, administration of MEDI4893 has been shown to improve survival in an immunocompromised mouse pneumonia model induced by cyclophosphamide [29], supporting the therapeutic potential of toxin neutralization in vulnerable hosts. For these individuals, vector-mediated antibody delivery may offer a promising alternative to vaccination, and could potentially provide durable passive immunoprophylaxis and long-term protection.

During staphylococcal sepsis, decreased platelet counts are a strong negative predictor of patient survival [45]. Platelets play a central role in disease progression by driving vascular occlusion through dysregulated aggregation and microthrombus formation, which contribute to multi-organ dysfunction and high mortality. In *S. aureus* sepsis, platelet activation and depletion are particularly pronounced. We previously demonstrated that AT binding to platelets promotes vascular occlusion via platelet activation and aggregation [17], with recent work showing that endothelial expression of ADAM10 is critical for AT-mediated lethality by further enhancing microthrombus formation [46]. Despite the detrimental effects of platelet dysfunction, accumulating evidence indicates that platelets also play protective roles in infection, including direct microbial killing, facilitating bacterial capture by liver Kupffer cells, and through neutrophil extracellular traps [17,47–49]. Unlike many antiplatelet drugs, neutralization of AT with serum antibodies preserves these beneficial platelet functions while preventing pathological aggregation, reinforcing its value as a therapeutic target. Moreover, AT activity extends beyond innate immunity, as increasing evidence suggests that it also modulates adaptive immune responses, including those of T and B cells [50].

In our pneumonia model, AAV-mediated delivery of MEDI4893 reduced sickness behavior and mortality without altering bacterial burden, underscoring the role of AT as a major driver of pathology rather than bacterial replication. This observation is consistent with prior studies showing that AT disrupts alveolar epithelial and endothelial barriers, impairs immune cell function, and contributes to lung injury during *S. aureus* pneumonia [51,52]. Notably, previous studies using purified MEDI4893 reported reductions in bacterial burden at 24 h post-infection [29,53], whereas we did not observe this effect. Interestingly, work from the Brönstrup laboratory demonstrated that chemical inhibition of AT with a small molecule protected mice from lethal pneumonia and, in contrast to our findings, also reduced bacterial burden in the lungs [18]. This discrepancy suggests that different modes of AT neutralization, antibody-based versus small-molecule inhibition, may differentially influence host–pathogen interactions, with small molecules potentially achieving better penetration into the lung, whereas we observed only limited distribution of MEDI4893 in bronchoalveolar lavage fluid.

Our findings demonstrate that AT is essential for establishing skin infection, as AAV-mediated delivery of MEDI4893 reduced bacterial burden and prevented dermal necrosis in a murine SSTI model. This is consistent with previous studies identifying AT as a major virulence factor in SSTIs. For example, Sampedro *et al.* (2014) reported that AT-deficient *S. aureus* mutants displayed reduced virulence in skin infection models, with markedly diminished necrosis and inflammation [28]. Likewise, Kennedy *et al.* (2010) showed that antibody-mediated neutralization or genetic deletion of AT significantly reduced lesion size and tissue injury [29]. In addition, the potential for resistance through mutation of the MEDI4893 epitope may be inherently limited, as previous work has shown that such mutations reduce AT activity and virulence [54], suggesting a fitness trade-off for escape variants. Importantly, MEDI4893 does not enhance opsonophagocytosis, as AT is a secreted toxin rather than a surface antigen; instead, protection arises from blocking AT-driven cytotoxicity that disrupts epithelial barriers and promotes bacterial persistence. By preserving epithelial integrity and immune function, MEDI4893 limits both tissue destruction and bacterial expansion in the skin, highlighting AT as a central virulence factor in SSTIs and supporting anti-toxin strategies as a therapeutic avenue.

Despite decades of effort, numerous vaccination strategies against *S. aureus* have failed to demonstrate efficacy in clinical trials, including those targeting capsular polysaccharides, iron-regulated surface determinants (IsdB), and multicomponent protein vaccines [55,56]. One reason for these failures may be the complexity of *S. aureus* immune evasion strategies and the redundancy of its virulence factors. A recent study by Tsai *et al.* further highlighted that pre-existing immunity from early-life exposure to *S. aureus* can actively compete with and undermine protective responses induced by vaccination [57]. This underscores the need for alternative approaches that bypass these barriers. By harnessing AAV6.2FF-mediated delivery of MEDI4893, we propose a preventative strategy that combines the durability of *in vivo* antibody expression with the proven efficacy of AT neutralization. This approach not only addresses the shortcomings of traditional vaccination but also offers a long-lasting prophylaxis option for high-risk populations. Looking ahead, combination therapies pairing AT neutralization with conventional antibiotics may further enhance protection, tackling both bacterial viability and virulence. Collectively, these data point to vector-mediated anti-toxin prophylaxis as a powerful and innovative strategy to prevent *S. aureus* infections.

## Materials and methods

### Ethics statement

Experiments performed at the University of Guelph were approved by the institutional animal care committee at the University of Guelph (AUP #4664), and experiments performed at the University of Calgary were approved by the University of Calgary Animal Care Committee (AC24-011). All experiments were conducted in accordance with the Canadian Council on Animal Care.

### Mice

Animal experiments were performed with equal groups (unless stated otherwise) of male and female 6 – 12-week-old Balb/c mice (purchased from Charles River Laboratories (Saint Constant, QC)) or C57/Bl6 mice (the Jackson Laboratory). Mice were housed at specific pathogen-free facilities under standardized conditions of illumination (12 h light/12 h darkness) and temperature (21–22 ˚C).

### Staphylococcal strains and culture conditions

Our laboratory strain of *S. aureus* USA300 with constitutive lux expression was created by transducing the *luxBACDE* locus encoded on plasmid pRP11995 that was previously integrated into *S. aureus* NRS384 (also known as USA300–0114) [58] using a lysate of phage 80⍺ and selection on chloramphenicol (10 µg/mL). Luminescence of the resulting transductants was confirmed and a representative strain was cryopreserved. For infections, staphylococci were grown overnight in brain heart infusion (BHI) (BD Bioscience) with or without 10 µg/mL chloramphenicol (BioShop) at 37˚C while shaking. Before starting the experiment, bacteria were subcultured for 2h at 37˚C while shaking to get into the exponential growth phase. Bacteria were washed with saline (Braun) and brought to a concentration of OD_660_ = 1.0, equivalent to 1x10^9^ CFU/mL from which bacteria were diluted to the appropriate concentration for infection.

### Cloning the MEDI4893 transgene

InFusion cloning (Takara; 639650) was used to clone the full-length MEDI4893 mAb gene into the modified AAV genome. The MEDI4893 genes were human-mouse codon-optimized and synthesized with human IgG1 heavy chain and human kappa light chain constant domains separated by an F2A self-cleaving peptide (see **Fig 1A** for schematic). Sourced from a gene block (synthesized by Genscript; U136EIC280), the MEDI4893 sequence was inserted downstream of the composite CASI promoter in an AAV genome containing AAV2 ITRs, a WPRE and an SV40 polyA tail. After cloning, transformation into NEB Stable competent *Escherichia coli* (*E. coli*) cells (New England Biolabs; C3040H) was performed and colonies were selectively grown on 50 µg/mL carbenicillin plates. Eight colonies were chosen for DNA isolation using Invitrogen Mini prep kit and a confirmatory digest with HindIII, BamHI, and NheI restriction enzymes was performed to compare the desired plasmid to the re-ligated backbone. After the confirmatory digest, the integrity of the AAV-MEDI4893 plasmid was confirmed through whole-plasmid sequencing performed by Plasmidsaurus.

### Production of AAV6.2FF vector

For production of AAV6.2FF vectors, HEK293 (ATCC; CRL-1573) cells were cultured in DMEM (Fisher Scientific; SH30022FS) with 8% Cosmic Calf Serum (Fisher Scientific, SH3008703HI), 2% penicillin streptomycin (Fisher Scientific; SV30010), and 2 mM L-glutamine (Fisher Scientific; SH3003402). The day prior to transfection, forty 15 cm tissue culture plates (Corning; 430599) were seeded at 5x10^6^ cells per plate. 16–18 hours post-seeding, cells were transfected at an optimal confluency of 80–90%. A dual plasmid transfection method was used with a 1:3 transgene to pDGM6.2FF ratio, and a total of 40 μg DNA per plate. A 3:1 ratio of PEI Max (PolyBiosciences; 24765-1) to total DNA was used, and the mixture was topped up to a total volume of 2 mL/plate with OptiMEM (Fisher Scientific; 31985-070). After the PEI Max was added to the DNA/Opti-MEM mixture, it was incubated for 10 minutes at room temperature before adding 2 mL to each plate of cells.

Supernatant and cells from transfected plates were harvested four days post-transfection, followed by ultracentrifugation and cell lysis to collect rAAV that was not secreted. AAV6.2FF was purified and concentrated using a Cytiva HiTrap Heparin HP Column (Cytiva; 17040703).

Each preparation of AAV6.2FF-MEDI4893 was examined for quality and concentration of vector genomes. Alkaline gel electrophoresis was used to assess genome integrity, and SDS-PAGE and Coomassie protein staining was used to assess vector purity. Quantitative PCR (qPCR) was performed on a Roche LightCycler 480 to determine the physical titer of each batch of AAV6.2FF-MEDI4893. As a control, we produced a vector expressing CA45, a monoclonal antibody specific against Ebola virus [32] according to the method described above.

### Evaluation of MEDI4893 expression *in vivo*

1x10^11^ vector genomes (vg) of AAV6.2FF-MEDI4893 were diluted to a total volume of 40 μL PBS and administered intramuscularly to 6-week-old female Balb/c pathogen-free mice. Mice underwent saphenous vein bleeds weekly for 5 weeks post AAV6.2FF-MEDI4893 injection. Mice also underwent terminal bleeds upon euthanasia at 6 weeks post AAV6.2FF-MEDI4893 injection. Blood was collected in EDTA Capillary blood collection tubes (FisherScientific; NC9141704) and spun at 5000 rpm for 5 minutes to collect plasma. Plasma from blood was run on a Human IgG ELISA kit (Abcam; ab195215) to quantify serum MEDI4893 expression levels at each time point.

Six weeks post AAV6.2FF-MEDI4893 injection, mice were euthanized by isoflurane anesthesia and CO_2_ overdose. Lavages were performed to assess human IgG levels at mucosal surfaces. Bronchioalveolar lavages (BAL) were performed by inserting a 22G x 1” catheter into the trachea and slowly dispensing then withdrawing 800 μL of PBS. Peritoneal lavages were performed by injecting 1 mL of PBS into the peritoneal cavity and massaging the abdomen before withdrawing the fluid. Vaginal lavages were performed by rinsing the vaginal canal with 50 μL of PBS three times for a total of 150 μL of lavage fluid. Intestinal lavages were performed by removing 10 cm of the small intestine and pushing 1 mL of PBS wash through, collecting the lavage fluid in a petri dish. All lavage samples were clarified at 1500 g for 5 minutes.

### Alpha-toxin binding assay

Commercially available human IgG ELISA kits (Abcam 195125) were used to determine concentrations of human immunoglobulin in serum and lavage fluid. A functional binding ELISA was performed to confirm binding of vectorized MEDI4893 antibody to AT. AT was a kind gift from Dr Sellman and recombinantly produced as described [30]. Half-well 96-well plates (Corning 3690) were coated with 1 μg/mL AT diluted in coating buffer (0.1 M bicarbonate buffer, pH 9.5) overnight at 4˚C. Plates were blocked with SuperBlock Blocking Buffer in PBS (ThermoFisher, 37515) for 60 minutes at 37˚C with gentle agitation. Plates were washed three times with 0.2% Tween-20 in PBS, and 30 µL of serum samples diluted in SuperBlock were added to the wells and incubated at 37˚C for 60 minutes with gentle agitation. The plates were washed three times with 0.1% Tween-20 in PBS. Goat anti-human HRP conjugated secondary antibody diluted 1:3000 in SuperBlock was added and plates were incubated at 37˚C for 60 minutes with gentle agitation. Plates were washed three times with 0.2% Tween-20 in PBS and incubated at room temperature for 15 minutes with TMB substrate. Optical Density (OD) values at 600 nm were recorded and graphed after subtraction of the mean OD of the negative control.

### Toxin-mediated cell death assay

A549 cells (type 2 alveolar-like epithelial cells) were seeded at 2x10^4^ cells per well in a 96-well plate in Ham’s F-12K (Kaighn’s) Medium with 10% FBS. Subsequently they were cultured for 48 hours at 37°C with 5% CO_2_. Mouse serum from CA45 or MEDI4893 infected mice was serially diluted. Equal volumes of serum and 1000ng/mL of recombinant α-toxin were mixed and incubated for 30 minutes at room temperature with gentle rocking. Meanwhile, the A549 cells were washed once with 100ul Ham’s F-12K (Kaighn’s) Medium per well and subsequently incubated in Ham’s F-12K (Kaighn’s) Medium with 2% FBS containing a final concentration of 0.4 µg/mL propidium iodide (PI). The serum-toxin solution was then added to the cells and images were acquired at 12 and 24 hours post-treatment using a Leica Stellaris 5 confocal microscope. PI-positive cells were quantified using the LAS X software (Leica Microsystems), and cell death was calculated by normalizing to wells treated with 1000 ng/mL of AT alone using the following formula:


$$\text{Percentage of Cell Death in Comparison to Alpha Toxin}=\frac{{\text{PI}}_{\text{serum}+\text{toxin}}}{{\text{PI}}_{\text{toxin only}}}\times \text{100}$$

### Alpha-toxin challenge studies

3-week-old male and female C57/Bl6 mice were intramuscularly injected with 1x10^11^ vg of AAV6.2FF-MEDI4893 diluted in a total volume of 25 µL PBS. 3 weeks post vaccination, mice were anesthetized with an intraperitoneal injection of ketamine (200 µL/gr, Vetoquinol) and xylazine hydrochloride (10 µL/gr, Dechra). A catheter was inserted in the tail vein to gain access to the bloodstream. 1 µg AT was administered to mice injected with PBS or mice injected with AAV6.2FF-MEDI4893. Mice were closely monitored over a period of 4 hours for vital signs, and the humane endpoint was dictated by the murine sepsis score system which considers appearance, consciousness, activity, response to stimuli, respiration rate and quality (**S1 Table**). Each criterion was graded 1–4 points and a score of 4 in any criterion or a total score higher than 18 was considered a humane endpoint and the animal was sacrificed via cervical dislocation under anesthesia. Prior to AT administration and 10 minutes post AT injection 50 µL of blood was collected through retro-orbital bleeding. Blood samples were stored in EDTA tubes (EDTA KE/1.3, Sarstedt) at room temperature and placed in an automated Hematology Analyzer (VetScan HM5, Zoetis) to quantify platelet numbers.

### Recombinant expression of alpha-toxin production and labelling

The AT gene of *S. aureus* strain Newman was synthesized and modified by the addition of a C-terminal cysteine, followed by a thrombin cleavage site and a His-tag. This was cloned into the pET28A vector (Novagen) and transformed into *E. coli* BL21(DE3) (Thermo Scientific). For a typical sample preparation, bacteria were grown in Luria Broth at 37 °C until A_600_ of 0.5, prior to addition of 1 mM isopropyl β-d-thiogalactopyranoside to induce protein expression. After 4 hours, cells were harvested by spinning at 4 °C for 15 minutes at 3500 × *g*. The supernatants were discarded, and pellets were directly lysed through resuspension in a denaturing lysis buffer (50 mM Tris, 500 mM NaCl, 6 M Guanidine hydrochloride, pH 8) and sonification. His-tagged AT was isolated using 5 mL HisTrap HP columns (GE Healthcare) according to manufacturer’s protocol. To label the purified AT, it was incubated with 10 × molar excess of Alexa Fluor 647-C2-Maleimide (Invitrogen Cat #A20347) at room temperature for 2 hours. The reaction was quenched with 0.01 M gluthathion. Excess dye and gluthathion were removed through extensive dialysis into PBS.

### Spinning-disk intravital microscopy

Platelet aggregation and colocalization were assessed through spinning-disk intravital microscopy (SD-IVM). Mice were anesthetized with an intraperitoneal injection of ketamine (200 µL/gr, Vetoquinol) and xylazine hydrochloride (10 µL/gr, Dechra). A catheter was inserted in the tail vein to gain access to the bloodstream. A laparotomy was performed as previously described [59] to prepare the mouse for intravital imaging of the liver. Mouse body temperature was maintained at 37 ˚C by means of a heated surgery plate (Live Cell Instrument) as well as a heated microscopy stage (Quorum). Imaging was performed on a Nikon Ti2-E X1 inverted microscope equipped with a CSU spinning disk system (Yokogawa) and ORCA-Fusion BT sCMOS camera (Hamamatsu). Target cells were visualized using fluorescent antibodies at 2.5 µg/mouse. Platelets were stained with AF647 or PE anti-mouse CD49b (BioLegend clone: HMa2) and Kupffer cells were stained with BV421 anti-mouse TIM-4 (BD clone: 21H12). Laser excitation wavelengths 405, 488, 561, and 640 nm (Nikon LUNF) were used in rapid succession and passed through the appropriate band-pass filters (Chroma). Videos were acquired at 6 random fields of view with 10X air or 20X water objective for 25 minutes. After one minute of imaging, AT or AF647-C20N aleimide-labelled AT were injected intravenously into mice. Images and Z stacks were acquired with 10X air, 20X water, or 40X water objectives. NIS analysis software (version 5.42.04) was used for 3D rendering, visualization, and analysis of the acquired images. Platelet aggregation was quantified by measuring fluorescent intensity of the platelet signal over time as well as the number of platelet aggregates per field of view. For visualization purposes, some videos for publication were subjected to the AI denoise function of the software.

### Lung infection model

*S. aureus* USA300 LAC bacteria were cultured as described previously and brought to a concentration of 5x10^8^ CFU/40 µL. C57/Bl6 mice were anesthetized with 4% isoflurane (Fresenius Kabi) until deep breathing occurred. Mice were taken from the isoflurane chamber and infected intra-tracheally with 5x10^8^ CFU bacteria. Covering the nose of mice as well as holding the tongue ensured for breathing in of bacteria for 15 gasps of breath. Mice were closely monitored daily for 4 days for weight changes and vital signs. The humane endpoint was dictated by the murine sepsis score system which considers appearance, consciousness, activity, response to stimuli, respiration rate and quality as described previously (**S1 Table**). Weight loss exceeding 20% of starting weight as well as a score of 4 in any criterion or a total score of higher than 18 was considered as a humane endpoint and the animal was sacrificed via cervical dislocation under anesthesia.

### Bacteriological analysis

The right lobes of the lung, as well as the spleen, a piece of liver, and left kidney were put into 1 mL PBS in tubes that were weighed before and after addition of the organ to get the weight of the lung. Tissues were homogenized using a tissue homogenizer (VWR 200 Homogenizer) and diluted 1:10, 1:100, 1:1000, and 1:10000 in PBS. 30 µL of each dilution was plated on a single BHI plate to create four streaks of bacteria. Blood was collected and 25 µL of each sample was spread undiluted on a BHI plate. Plates were incubated overnight at 37˚C. Bacterial colonies were counted in the dilution showing 30 – 100 colonies and logCFU was calculated to determine the bacterial burden per gram of tissue.

### Cytospin on BAL fluid

After 24 hours of intratracheal infection, mice were sacrificed and BAL fluid was collected by inserting a 20G x 1” catheter (Terumo) into the trachea and slowly dispensing then withdrawing a total of 4 mL of HBSS + 10 mM EDTA approximately 700 µL at a time. After collection, any mucus clumps were removed by passing the sample through a 40 µm cell strainer (VWR). BALF was centrifuged at 1500 rpm for 5 minutes to separate the cells from the supernatant. Supernatant was removed and cells were taken up in 1 mL HBSS + 10 mM EDTA. 10 µL of cell suspension was diluted in 10 µL Trypan Blue Stain (0.4%; gibco) and counted with the Luna II automated cell counter. Then, 100 µL of cell suspension was spun on a Superfrost Plus Microscope Slide (Fisher Scientific) using cytospin. Slides were airdried for 2 hours before staining with the Hemacolor Rapid staining kit according to kit instructions (Sigma-Aldrich). Slides were dried for 1 hour before imaging with brightfield microscopy (Olympus BX51). 20 field of view images were taken at 20X magnification per slide. Immune cells were then quantified per field of view image.

### BCA assay on BALF supernatant

BAL fluid protein concentrations were quantified using the Pierce Rapid Gold bicinchoninic acid assay (BCA) kit (Thermo Scientific, Cat#A53225). The procedure was followed as per the manufacturer’s instructions. For SDS-PAGE analysis, 40 µL of each BAL fluid sample was mixed with 20 µl of 2x Laemmli sample buffer and then boiled for five minutes at 95°C. A total of 12.5 µL of each sample was loaded onto a 4–20% SDS-PAGE gel (Bio-Rad, Cat#4568096) and separated at 200 V for 35 minutes. Proteins were visualized with Coomassie Brilliant Blue G-250 stain (Fischer Scientific, Cat# ICN808274).

### Skin infection model

Luminescent *S. aureus* USA300 LAC bacteria were cultured as described above and brought to a concentration of 2x10^7^ CFU/50 µL. C57/Bl6 mice were anesthetized with isoflurane (5% induction, 3% maintenance, Fresenius Kabi) until reflexes were absent and the dorsal and lateral sides of the animals were shaven with a clipper. In each dorsal flank a dose of 2x10^7^ CFU in 50 µL PBS was administered subcutaneously. Mice were monitored daily for bacterial burden and dermal lesions. The dermal necrosis-related area was determined by measuring the size of the individual lesions with a ruler after daily digital pictures (Canon EOS Rebel T6) of the animals and dorsal skin area were taken and the area of dermal necrosis was calculated using ImageJ software.

Bacterial burden was determined longitudinally via an *in vivo* optical noninvasive whole-body imaging system (AMI HTX, Spectral Instruments Imaging, a BRUKER company). Animals were anesthetized with isoflurane (5% induction, 3% maintenance) and placed on a heated stage in the imaging system, exposing the dorsal side of the mice to capture infection-related bioluminescence (60s exposure time). Data was analyzed post-acquisition using system-related imaging software (AURA 4.5.1, Spectral Instruments Imaging, a BRUKER company). *S. aureus*-associated bioluminescence expression in the dorsal infected areas was quantified by measuring the total emission (photon/s) in a defined region of interest (ROI) resembling the outline of the lesion areas each day in each animal.

### Statistical analysis

Statistical analysis was performed using GraphPad Prism (v11) software. For comparison between two groups, the unpaired Student’s t-test was used. For comparisons between two groups over multiple timepoints, multiple Mann-Whitney tests were performed according to the Holm-Šídák method. Comparisons performed over three groups were tested with a Kruskal-Wallis test with multiple comparisons. The Kaplan-Meier test was performed together with a Log-rank (Mantel-Cox) test for survival curve analysis.

## Supporting information

S1 Fig Protection of cell death by mouse serum. Percentage of cell death of type 2 alveolar-like epithelial cells after addition of 100 ng/mL AT with 10% mouse serum of either AAV-CA45- or AAV-MEDI4893-treated mice (*n* = 5). Serum was collected from mice infected with *S. aureus* (5x10^8^ CFU, intratracheal infection). Datapoints are normalized against a 1000 ng/mL AT only condition. Mann-Whitney test, mean + /- SD.
(TIFF)

S2 Fig Decreased platelet numbers after α-toxin challenge. Platelet counts measured at baseline (pre-AT infusion) and 10 min post-AT infusion in untreated control animals and AAV6.2FF-MEDI4893-treated mice. (*n* = 4 per group); multiple Wilcoxon tests, data not significant.
(TIFF)

S3 Fig Weight measurements after *S. aureus* USA300 pneumonia. Monitoring of weight change after intratracheal infection with *S. aureus* USA300 (5x10^8^ CFU) over a period of 4 days. *n* = 8 for untreated control mice and *n* = 7 for AAV6.2FF-MEDI4893 treated mice. Multiple Mann-Whitney tests, data not significant.
(TIFF)

S4 Fig Total protein levels from bronchoalveolar lavage fluid samples. SDS-PAGE depicting the various protein levels in BAL fluid samples isolated from the mice in Fig 3H. Separated proteins were visualized with Coomassie Brilliant Blue stain.
(TIFF)

S5 Fig Bacterial dissemination to other organs after *S. aureus* USA300 pneumonia. Bacterial burden in blood, spleen, liver, and kidney tissues at 24 h post intratracheal infection with *S. aureus* (5x10^8^ CFU USA300 LAC). (*n* = 6 for AAV6.2FF-CA45 treated group and *n* = 5 for AAV6.2FF-MEDI4893 treated group), Mann-Whitney test, data not significant; median.
(TIF)

S1 Table Sepsis scoring system. Scoring system used to create a sepsis score during survival study of *S. aureus* pneumonia. This system considers appearance, consciousness, activity, response to stimuli, and respiration rate and quality. Every component is given a score between 1 and 4 and a score of 4 in any one criterion or a cumulative score > 18 was defined as a humane endpoint.
(DOCX)

S2 Table Raw data. Minimal dataset table showing all raw data values for each graph in the manuscript.
(XLSX)

S1 Video Platelet aggregation after α-toxin challenge in untreated control mouse. Representative 10× SD-IVM video of a murine liver over 20 min. At 1 min, 4 µg α-toxin (AT) was injected intravenously. Platelets (CD49b-AF746, red) rapidly aggregated in liver sinusoids. Hepatocytes (autofluorescence, dull green) and Kupffer cells (TIM-4–BV421, white) are shown.
(MP4)

S2 Video Absence of platelet aggregation after α-toxin challenge in AAV-MEDI4893–treated mouse. Representative 10× SD-IVM video of a murine liver over 20 min. At 1 min, 4 µg AT was injected intravenously. Platelets (CD49b-AF647, red) moved freely through liver sinusoids throughout the recording. Hepatocytes (autofluorescence, dull green) and Kupffer cells (TIM-4–BV421, white) are shown.
(MP4)

S3 Video Visualization of fluorescently labeled α-toxin binding to platelets. Representative 10 × SD-IVM video of a murine liver over ~10 min. At 1 min, N-terminally labeled α-toxin (AT-AF647, blue) was injected intravenously, producing a transient fluorescent flush through liver sinusoids followed by binding to platelets (CD49b-PE, red) and subsequent platelet aggregation. Kupffer cells (TIM-4–BV421, white) are also shown.
(MP4)
