# Supplementary figures and images for: Adeno-associated virus delivery of anti-alpha toxin monoclonal antibodies confers protection against Staphylococcus aureus infections
Source: PLoS Pathog. 2026 Apr 6;22(4):e1014090. doi: 10.1371/journal.ppat.1014090 (PMC13089897; doi:10.1371/journal.ppat.1014090)

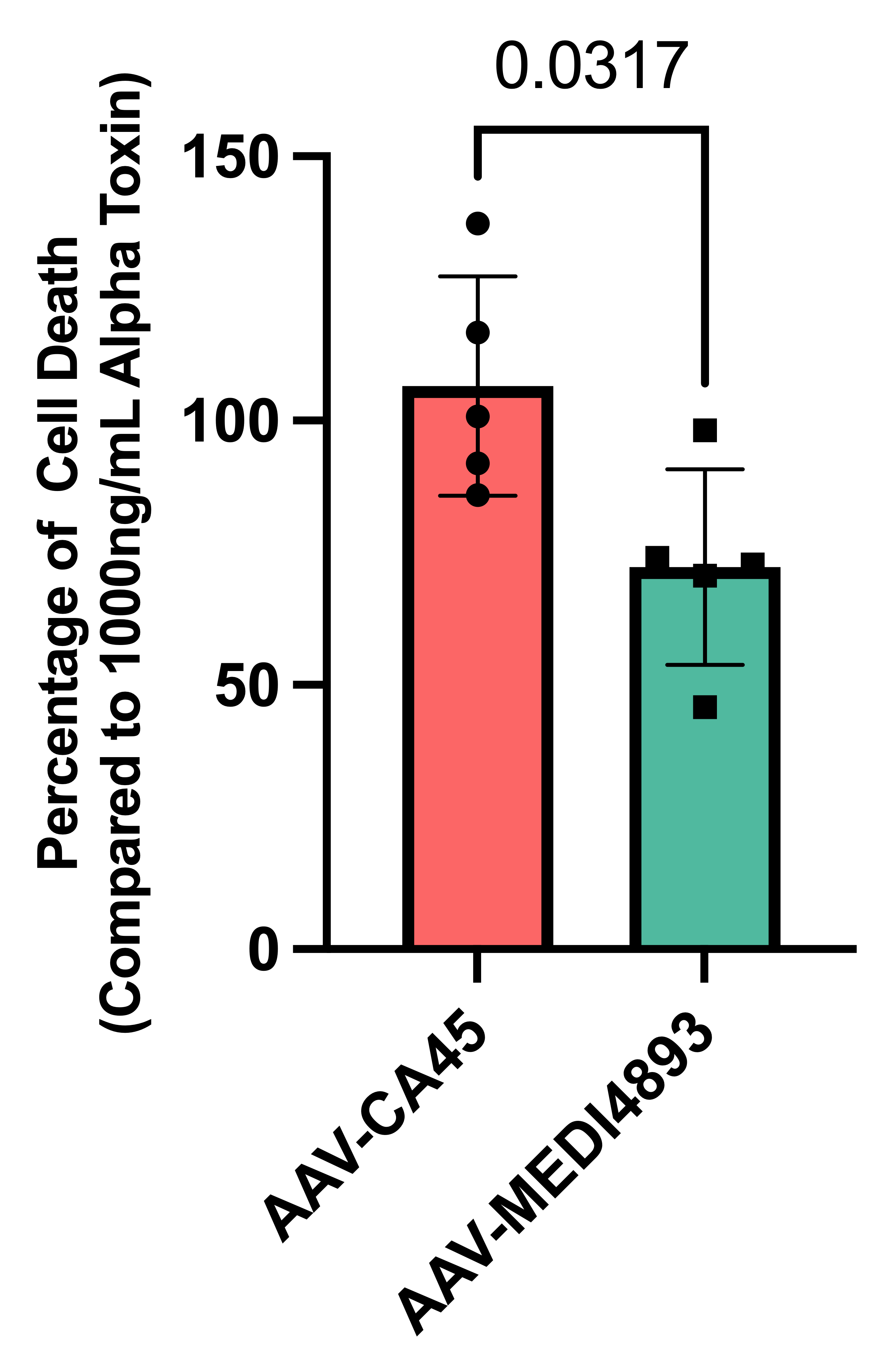

Supplement: S1 Fig — Percentage of cell death of type 2 alveolar-like epithelial cells after addition of 100 ng/mL AT with 10% mouse serum of either AAV-CA45- or AAV-MEDI4893-treated mice (n = 5). Serum was collected from mice infected with S. aureus (5x10^8^ CFU, intratracheal infection). Datapoints are normalized against a 1000 ng/mL AT only condition. Mann-Whitney test, mean + /- SD. (TIFF) [file ppat.1014090.s001.tiff]

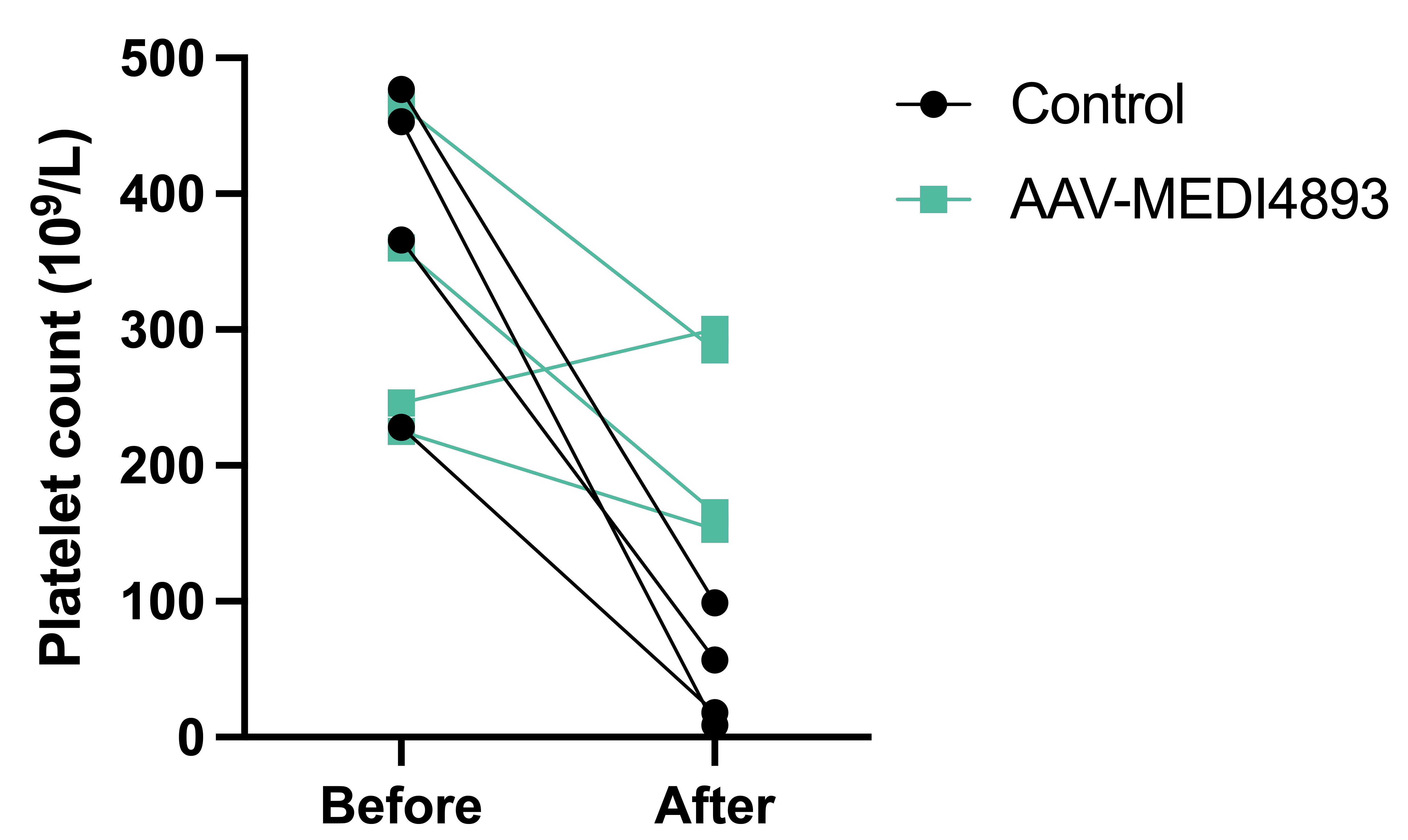

Supplement: S2 Fig — Platelet counts measured at baseline (pre-AT infusion) and 10 min post-AT infusion in untreated control animals and AAV6.2FF-MEDI4893-treated mice. (n = 4 per group); multiple Wilcoxon tests, data not significant. (TIFF) [file ppat.1014090.s002.tiff]

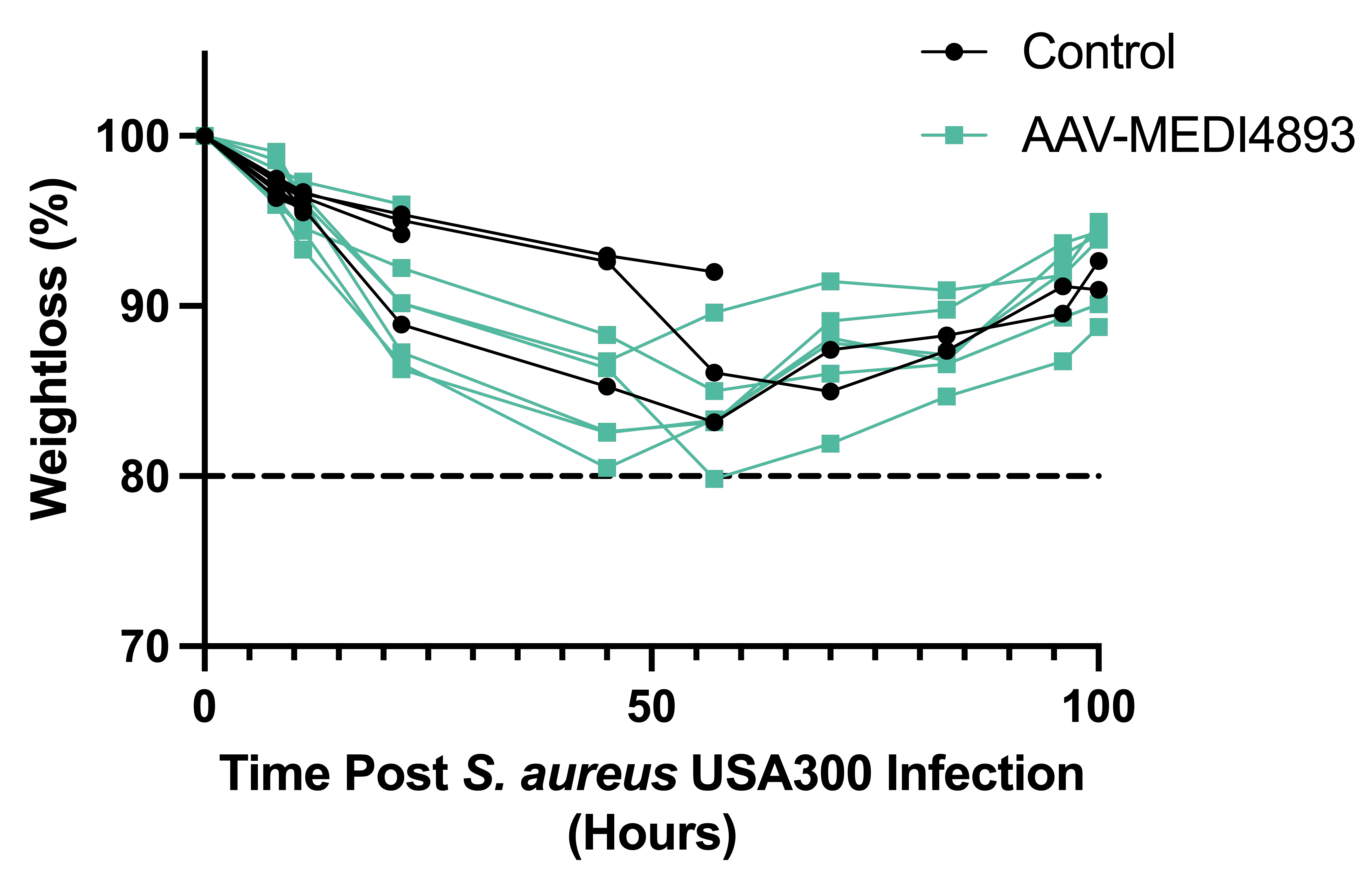

Supplement: S3 Fig — Monitoring of weight change after intratracheal infection with S. aureus USA300 (5x10^8^ CFU) over a period of 4 days. n = 8 for untreated control mice and n = 7 for AAV6.2FF-MEDI4893 treated mice. Multiple Mann-Whitney tests, data not significant. (TIFF) [file ppat.1014090.s003.tiff]

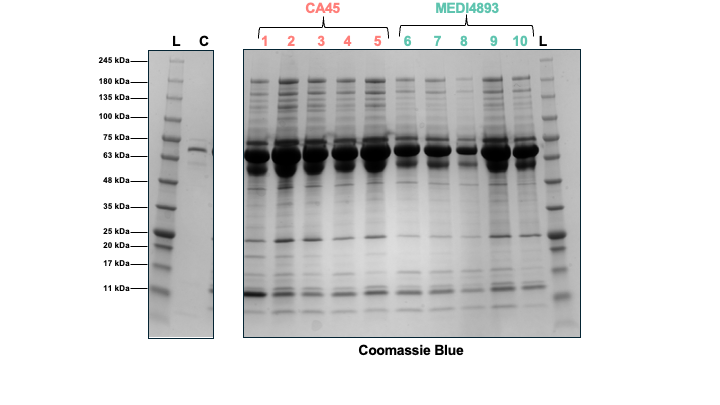

Supplement: S4 Fig — SDS-PAGE depicting the various protein levels in BAL fluid samples isolated from the mice in Fig 3H. Separated proteins were visualized with Coomassie Brilliant Blue stain. (TIFF) [file ppat.1014090.s004.tiff]

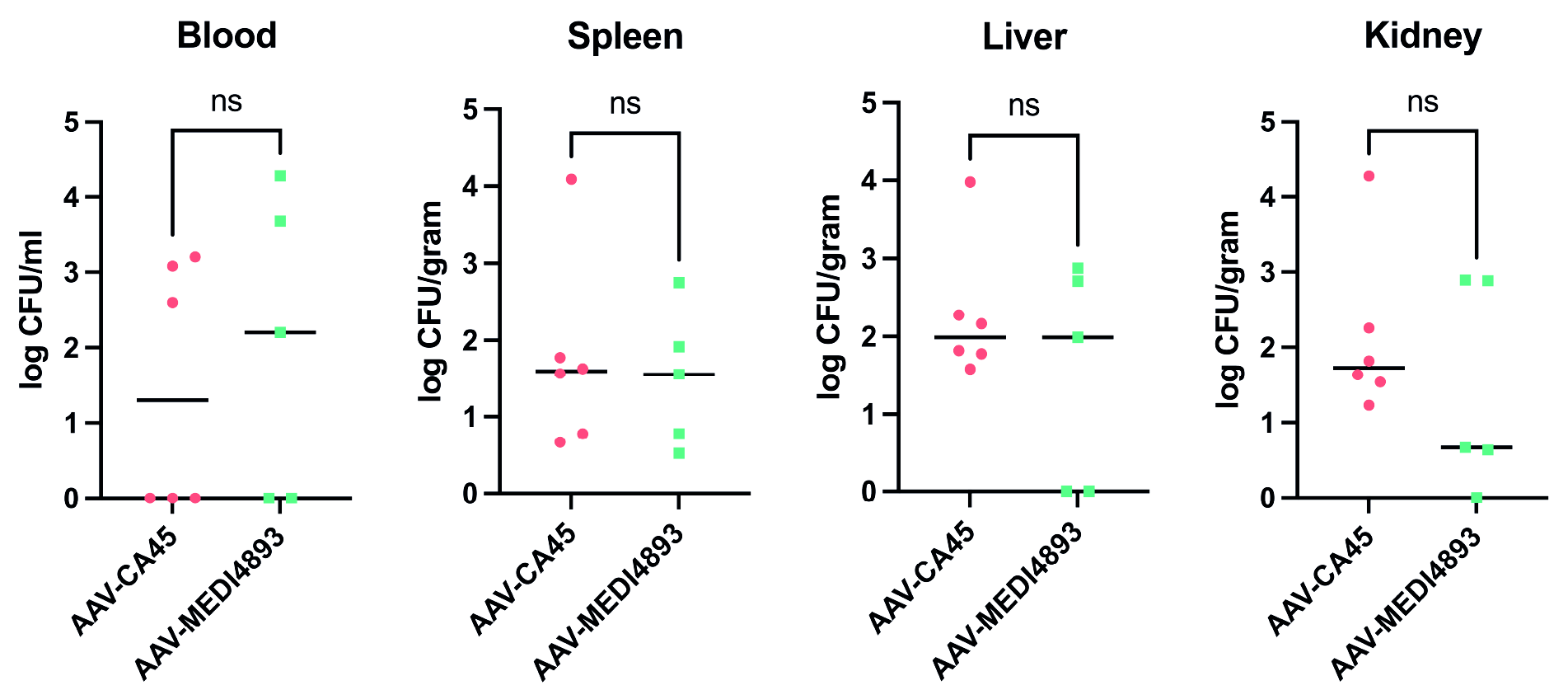

Supplement: S5 Fig — Bacterial burden in blood, spleen, liver, and kidney tissues at 24 h post intratracheal infection with S. aureus (5x10^8^ CFU USA300 LAC). (n = 6 for AAV6.2FF-CA45 treated group and n = 5 for AAV6.2FF-MEDI4893 treated group), Mann-Whitney test, data not significant; median. (TIF) [file ppat.1014090.s005.tif]
