# Supplementary material for: Adeno-associated virus delivery of anti-alpha toxin monoclonal antibodies confers protection against Staphylococcus aureus infections
Source: PLoS Pathog. 2026 Apr 6;22(4):e1014090. doi: 10.1371/journal.ppat.1014090 (PMC13089897; doi:10.1371/journal.ppat.1014090)
Supplement: S1 Table — Scoring system used to create a sepsis score during survival study of S. aureus pneumonia. This system considers appearance, consciousness, activity, response to stimuli, and respiration rate and quality. Every component is given a score between 1 and 4 and a score of 4 in any one criterion or a cumulative score > 18 was defined as a humane endpoint. (DOCX) [file ppat.1014090.s006.docx]

**Supplemental table 1. Sepsis scoring system.**

Scoring system used to create a sepsis score during survival study of *S. aureus* pneumonia. This system considers appearance, consciousness, activity, response to stimuli, and respiration rate and quality. Every component is given a score between 1 and 4 and a score of 4 in any one criterion or a cumulative score > 18 was defined as a humane endpoint.

| Sepsis Scoring System | |  |  |  |
| --- | --- | --- | --- | --- |
| **Score** | **1** | **2** | **3** | **4** |
| **Fur** | Grooming | Dull Coat | Rough Coat | Piloerection |
| **Activity** | Normal | Reduced when Disturbed | No Movement when Disturbed, Only Stimulated | No Activity |
| **Posture** | Normal | Slight Hunch | Hunched and Stiff | Hunched and not Moving |
| **Behavior** | Normal | Slow, Normal when Disturbed | Abnormal, Moves only when Disturbed | No Relocation, Very Abnormal |
| **Chest Movement** | Normal | Mildly Dyspneic | Moderately Dyspneic | Severely Dispneic, Abdominal Gasps |
| **Chest Sounds** | None | Occasional Chirp | Frequent Chirp | Wet Chirp |
| **Eyes** | Open | Near Closed but Open when Disturbed | Near Closed most of the time | Closed, Near Closed Stimulated |
